# Incidence of FGFR2 Amplification and FGFR2 Fusion in Patients with Metastatic Cancer Using Clinical Sequencing

**DOI:** 10.1155/2022/9714570

**Published:** 2022-03-18

**Authors:** Sujin Hyung, Boram Han, Jaeyun Jung, Seung Tae Kim, Jung Yong Hong, Se Hoon Park, Dae Young Zang, Joon Oh Park, Young Suk Park, Kyoung-Mee Kim, Won Ki Kang, Jeeyun Lee

**Affiliations:** ^1^Innovative Institute for Precision Medicine, Samsung Medical Center, Seoul, Republic of Korea; ^2^Division of Hematology-Oncology, Department of Medicine, Hallym University, Hallym Sacred Heart Hospital, Anyang, Republic of Korea; ^3^Division of Hematology-Oncology, Department of Medicine, Sungkyunkwan University, Samsung Medical Center, Seoul, Republic of Korea; ^4^Department of Pathology and Translational Genomics, Department of Medicine, Sungkyunkwan University, Samsung Medical Center, Seoul, Republic of Korea

## Abstract

Aberrations in the *fibroblast growth factor receptor2* (*FGFR2*) gene, including genetic alterations and chromosomal rearrangements, lead to the development and progression of cancer with poor prognosis. However, the mechanisms underlying the FGFR2 signaling pathway to facilitate the development of FGFR2-targeted therapies have not been fully explored. Here, we examined the clinicopathological features of *FGFR2* amplification and fusion in gastrointestinal tract/genitourinary tract cancers. *FGFR2* amplification and fusion were identified in approximately 1.5% and 1.1% of all cancer types in 1,373 patients, respectively, with both *FGFR2* amplification and fusion occurring together at a rate of approximately 0.6%. Of all cancer types screened, gastric cancer (GC) was the most common cancer type with *FGFR2* amplification (87.5% of all *FGFR2* amplification case) or fusion (46.7% of all cases). In addition, *FGFR2* alteration had poorer overall survival (OS, 13.7 months vs. 50.2 months, *P* = 0.0001) and progression-free survival (PFS, 5.6 months vs. 11.4 months, *P* = 0.0005) than did those without FGFR2 alteration, respectively. Taken together, our data underscore to screen solid cancer patients for FGFR2 aberrations in oncology clinic.

## 1. Introduction

Fibroblast growth factor receptor 2 (FGFR2), which belongs to a family of highly conserved tyrosine kinase receptors (FGFR 1–4), has emerged as a critical oncogenic factor that controls cancer development and progression [1–4]. However, the underlying mechanisms of action of FGFR2 are not fully understood [2, 5]. Under normal conditions, the FGFR system contributes to the regulation of several developmental processes, including the induction of organogenesis and homeostasis [4, 6]. Dysfunction of the FGFR system results in uncontrolled cell proliferation, migration, and survival, leading to cancer [4, 6, 7]. Although *FGFR2* aberrations, including gene amplification, fusion, mutation, and overexpression, have been detected in various types of cancer [3, 8–13], clinically available treatment options are limited.

Accumulating evidence indicates the significance of *FGFR2* in the tumorigenesis and progression of gastric cancer (GC); genomic aberrations in *FGFR2* have been linked to one of the most frequently occurring oncogenic aberrations [2, 14–17]. Although the incidence of *FGFR2* aberrations is relatively low, it is associated with a poor prognosis in GC [18]. Alterations in FGFR genes, such as amplification, mutation, and fusion, can lead to the aberrant activation of downstream components of FGFR-related pathways and induce mesenchymal transition as well as antiapoptotic responses in cancer cells [9, 19–21]. Based on recent studies involving selective pharmacological inhibition of FGFR2 in a model of FGFR2 amplification, this receptor is a promising therapeutic target in solid tumors, especially in GC [15, 17, 22].

Several clinical trials of FGFR-targeted therapy, including FGFR2, have been conducted in patients with GC [23–25]. In a recent phase 2 study, the addition of the anti-FGFR2 antibody to first-line chemotherapy demonstrated superior survival in FGFR2-overexpressed GC patient cohort (the FIGHT trial) when compared to chemotherapy alone [26].

We analyzed the clinicopathological features of *FGFR2* aberrations, including amplification and fusion, in patients with solid tumors who received clinical next-generation sequencing (NGS) as part of the clinical practice. In this study, we aimed to demonstrate the incidence and prevalence of FGFR aberrations using a clinical NGS platform.

## 2. Results

### 2.1. Patient Characteristics

**Figure 1:**
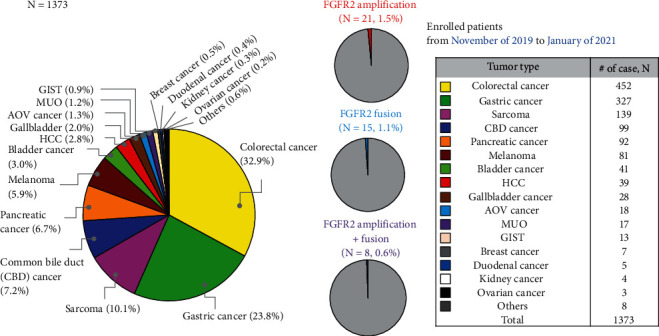
Overview of patients with cancer enrolled in the study and the proportions of fibroblast growth factor receptor 2 (*FGFR2*) genetic alterations. Between December 2019 and January 2021, patients with stage IV cancer were screened for *FGFR2* aberrations via next-generation sequencing using a panel that targeted 500+ genes in the Gastrointestinal/Genitourinary/Rare Cancer/Phase I Oncology Clinic of Samsung Medical Center. A Venn diagram indicating the (a) percentage of each type of cancer in a total of 1,373 patients and the (b) percentage of cases with *FGFR2* amplification, *FGFR2* fusion, or the cooccurrence of *FGFR2* amplification and fusion is shown. (c) A summary table showing the tumor types and numbers of patients is shown. AOV: ampulla of vater; CBD: common bile duct; HCC: hepatocellular carcinoma; MUO: metastasis of unknown origin; GIST: gastrointestinal stromal tumor.

Between November 2019 and January 2021, 1,373 oncology patients diagnosed with metastatic solid tumors received a clinical sequencing panel at Samsung Medical Center. NGS was performed with TruSight Oncology 500 (TSO500) assay (Illumina, San Diego, CA, USA) as described previously [27–29]. The panel includes multiple variant types across 523 genes and enables quantitative assessment of MSI and TMB status. The patient cohort consisted of 1,373 patients with 24 different types of solid tumors. The most common cancer types were colorectal cancer (CRC, 452 cases, 32.9%), GC (327 cases, 23.8%), and sarcoma (139 cases, 10.1%) (Figure 1). In all, 28 patients (*N* = 28, 2.0%) had FGFR2 aberrations detected in their tumor by NGS panel. Of the 28 patient, 21 (1.5%) patients had FGFR2 amplification, and 15 (1.1%) patients had FGFR2 fusion alone. Of note, 8 (0.6%) patients had both FGFR2 amplification and FGFR2 fusion in their tumor specimen.

### 2.2. *FGFR2* Amplification Was Predominantly Detected in Gastric Cancer

**Figure 2:**
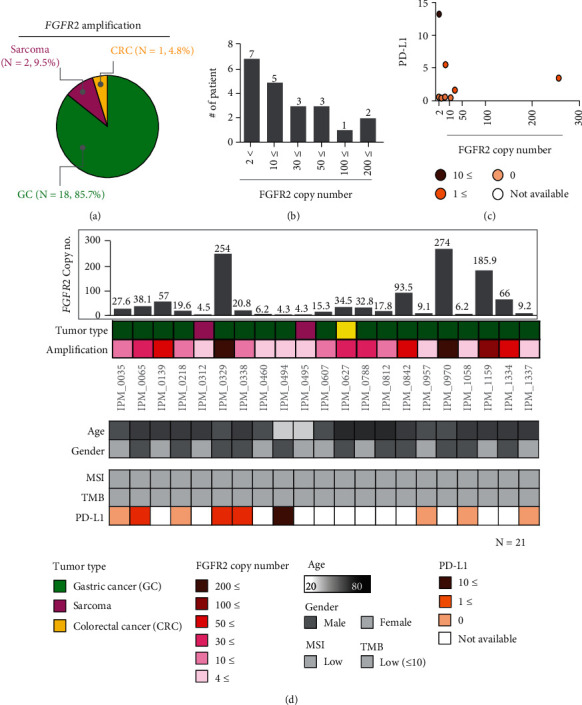
Clinicopathological landscape of patients with different cancer types exhibiting *FGFR2* amplification. (a) Venn diagram showing the percentage distribution of tumor types with *FGFR2* amplification (21 cases): GC (85.7%), sarcoma (9.5%), and CRC (4.8%). (b) *FGFR2* copy number. (c) Analysis of the correlation between PD-L1 level and *FGFR2* copy number. (d) Patient profiles, including the distribution of *FGFR2* copy number of all patients, tumor type and *FGFR2* amplification level (top), age and sex (middle), and MSI, TMB, and PD-L1 status (bottom). MSI: microsatellite instability; PD-L1: programmed cell death-ligand 1; TMB: tumor mutational burden.

Of the 21 patients with *FGFR2* amplification, 18 (85.7%) had GC, followed by sarcoma (*N* = 2, 9.5%) and CRC (*N* = 1, 4.8%). For GC patients, the incidence of *FGFR2* amplification, *FGFR2* fusion, and the cooccurrence of *FGFR2* amplification and fusion were 18 (5.5%), 7 (2.1%), and 7 (2.1%) patients, respectively. The median age of all patients was 48 years (range, 20–60 years) (Figures 2(a) and 2(d)). There were no cases of *FGFR2* deletion in our cohort. Of note, patients with *FGFR2* amplification had MSS and TMB low (less than 10 mutations/mB), indicating that *FGFR2* amplification predominantly occurs in the MSS subtype. In the FGFR2-amplification cohort (*N* = 28), the copy number ranged from 4.3 to 274 (Figure 2(b)). Six patients (28.5%) had over 50 copies of *FGFR2* in their tumor specimens. We performed immunohistochemistry against the 22C3 antibody to examine whether the *FGFR2* amplification was correlated with the level of PD-L1 expression. The combined positive score (CPS) for PD-L1, defined as the number of PD-L1^+^ cells, including tumor cells and immune cells (macrophages and lymphocytes), relative to the total number of tumor cells, is an important biomarker of cancer progression, with a CPS ≥ 1 considered a positive PD-L1 tumor. Of the 28 patients with FGFR2 aberration, 9 patients had PD-L1 data available. Of 9 patients, 5 patients had PD-L1 negative tumor while 4 patients had PD-L1^+^ tumor (Figures 2(c) and 2(d)).

### 2.3. The Incidence of *FGFR2* Gene Fusion with Various Partners in GC

**Figure 3:**
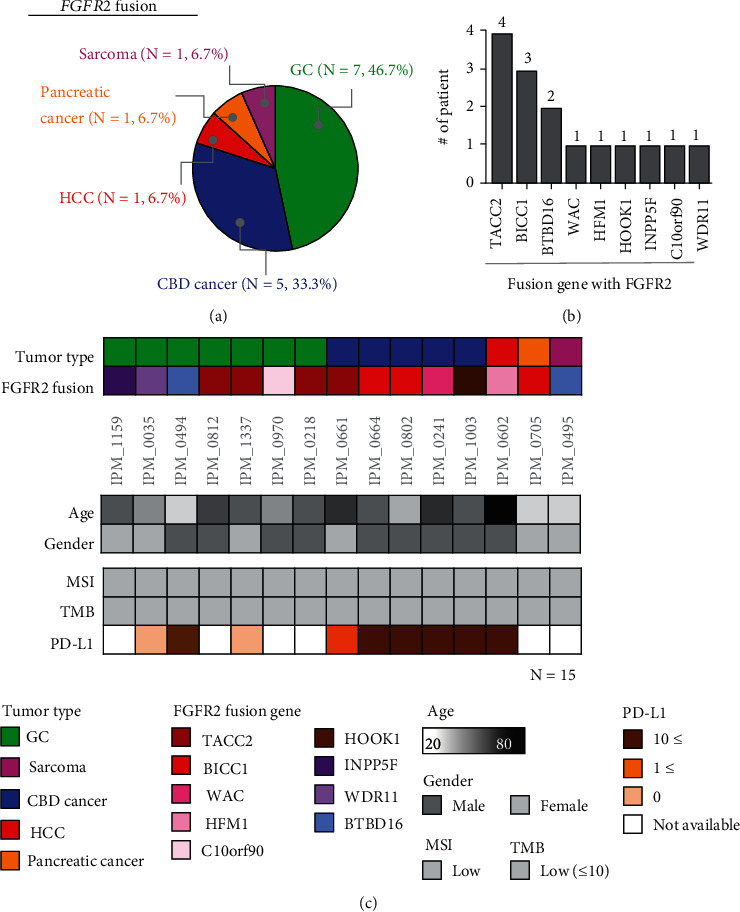
Clinicopathological landscape of patients exhibiting *FGFR2* fusion among all types of cancer. (a) Venn diagram showing the percentage distribution of tumor types with *FGFR2* fusion (15 cases): GC (46.7%), CBD cancer (33.3%), sarcoma (6.7%), pancreatic cancer (6.7%), and HCC (6.7%). (b) Patient profiles, including tumor type and *FGFR2* fusion partner genes (top), age and sex (middle), and MSI, TMB, and PD-L1 status (bottom). (c) *FGFR2* fusions with nine different partner genes were detected: *TACC2*, *BIVV1*, *BTBD16*, *WAC*, *HFM1*, *HOOK1*, *INPP5F*, *C10orf90*, and *WDF11*.

*FGFR2* fusions are the result of gene rearrangements and have been observed in different types of cancer with different incidence per cancer type [30–36]. Approximately 1.1% of the patients (15/1,373) had *FGFR2* fusions. The most common cancer types where FGFR2 fusion was detected were GC (*N* = 7, 46.7%), followed by common bile duct (CBD) cancer (*N* = 5, 33.3%), HCC (*N* = 1, 6.7%), pancreatic cancer (*N* = 1, 6.7%), and sarcoma (*N* = 1, 6.7%) (Figures 3(a) and 3(b)). The *FGFR2* fusion partners included *TACC2*, *BICC1*, *BTBD16*, *WAC*, *HFM1*, *HOOK1*, *INPP5F*, *C10orf90*, and *WDR11* (Figure 3(b), top panel). *FGFR2* was combined with the *TACC2* gene in 4 of 15 patients with FGFR2 fusion (26.7%). In addition, *BICC1-FGFR2* gene fusion was detected in 3 CBD and pancreatic cancer patients (3/15 cases, 20%), and *BTBD16-FGFR2* gene fusions were detected in 1 GC and 1 sarcoma patient (2/15 cases, 13.3%) (Figure 3(c)). Other *FGFR2* fusions were partnered with *WAC*, *HFM1*, *HOOK1*, *INPP5F*, *C10orf90*, and *WDR11* (Figure 3(c)). Similar to those with *FGFR2* amplification, all patients with *FGFR2* fusions had MSS and TMB-low status by NGS (Figure 3(b), bottom panel). Interestingly, there were 7 out of 9 patients with FGFR2 fusion and positive PD-L1 CPS. Interestingly, all 5 CBD cancer patients with FGFR2 fusion had PD-L1 positive tumor (Figure 3(b), bottom panel).

### 2.4. Cooccurrence of *FGFR2* Amplification and Fusion

**Figure 4:**
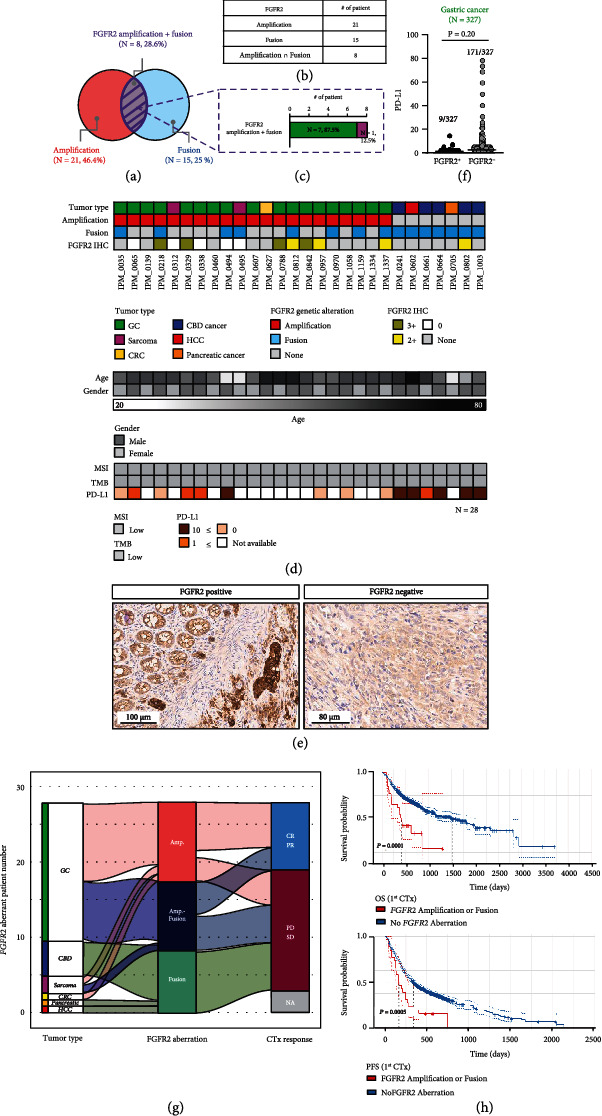
Genomic landscape of patients exhibiting cooccurrence of *FGFR2* amplification and fusion. (a) Venn diagram showing the percentage distribution of patients with *FGFR2* genomic alterations, including amplification (red), fusion (sky blue), and cooccurrence of amplification and fusion (purple) (*N* = 28). (b, c) Numbers of patients exhibiting *FGFR2* amplification, fusion, and cooccurrence of amplification and fusion and the percentage of cases with each tumor type: green, gastric cancer; magenta, sarcoma. (d) Comprehensive clinical characteristics of and the structural alterations present in patients, including tumor type, presence of amplification, presence of fusion, and FGFR2 IHC (top), age and sex (middle), and MSI, TMB, and PD-L1 status (bottom). (e) Representative immunohistochemistry results of FGFR2 showing strong cytoplasmic staining in the tumor cells. Scale bar: 100 *μ*m and 80 *μ*m. (f) Analysis of correlations between the PD-L1 expression level and *FGFR2* aberrations in GC (*N* = 327, FGFR2^+^: black circles and FGFR2^−^: gray circles). (g) Alluvial diagram representing the flow from tumor types to FGFR2 alteration and disease response to chemotherapy. (h) Overall survival (OS, top) and progression-free survival (PFS, bottom) curves calculated using the Kaplan–Meier method for groups classified according to FGFR2 alteration. CR: complete response; PR: partial response; SD: stable disease; PD: progressive disease.

Lastly, we surveyed the incidence of cancer patients with concurrent FGFR2 amplification and FGFR2 fusion in their tumor specimen. There were 8 patients with concurrent FGFR2 amplification and FGFR2 fusion (Figures 4(a), 4(b), and 4(d), top panel). Notably, approximately 87.5% of the cases with concurrent *FGFR2* amplification and *FGFR2* fusion occurred in GC patients (Figures 4(c) and 4(d)). Of the 28 patients with FGFR2 aberration, 15 patients had additional tissue specimens available for FGFR2 IHC. Of the 13 patients tested for FGFR2 IHC, 8 (61.5%) patients had FGFR2 overexpression by IHC (2+ in 4 patients and 3+ in 5 patients) (Figure 4(d)). Interestingly, three GC patients with concurrent *FGFR2* amplification and *FGFR2* fusion (3/7 cases, 42.8%) had FGFR2 protein overexpression in their tumor (Figure 4(d)). Representative image of FGFR2 protein overexpression by IHC is provided in Figure 4(e).

We next examined the correlation between PD-L1 expression and *FGFR2* genomic alterations (FGFR2^+^ or FGFR2^−^) in GC patient cohort (*N* = 327) (Figure 4). There were fewer FGFR2^+^ GC patients with PD-L1 expression (CPS ≥ 1) (4/9 cases, 44.4%) than those with a CPS indicating negative PD-L1 expression (5/9 cases, 55.6%), whereas FGFR2^−^ GC patients, including a substantial number of cases with a CPS, indicate positive PD-L1 expression (CPS ≥ 1; 120/171 cases, 70.1%) (Figures 4(d), bottom panel, and 4(f)). Specifically, 18 FGFR2− GC patients had high PD-L1 expression (CPS ≥ 20). However, the majority of GC patients with a CPS indicating positive PD-L1 expression exhibited low PD-L1 expression levels (CPS, 1–10) regardless of the presence or absence of *FGFR2* genetic alteration (approximately 75% for FGFR2^+^ and 68.9% for FGFR2^−^) (Figure 4(f)).

Among the 25 patients with *FGFR2* alteration assessable for treatment response, 64% of FGFR2 alteration flowed to PD/SD responses (*N* = 16), but 36% of FGFR2 alteration went to PR/SR responses (*N* = 9) (Figure 4(g)). Furthermore, the median overall survival (OS) among patients with FGFR2 alteration (13.7 months) was significantly shorter than that among those without alteration (50.2 months) (*P* = 0.0001). Similar results observed that the median progression-free survival (PFS) was 5.6 months and 11.4 months, respectively, in patients with and without FGFR2 alteration (*P* = 0.005) (Figure 4(h)). These results supported that FGFR2 alteration correlated with a poorer outcome.

## 3. Discussion

A number of studies have reported that *FGFR2* amplification is associated with GC development and progression, although the proportion of GC cases exhibiting *FGFR2* amplification is relatively low (up to ~5%) [2, 14–17]. Furthermore, high-level *FGFR2* amplification has been suggested to be associated with poor prognosis in several types of cancer, including GC [15, 16], CRC [14, 23], and breast cancer [5]. However, the clinical significance of *FGFR2* gene aberrations remains controversial, and further investigations to characterize *FGFR2* genetic alterations are needed. In this study, we showed that the rate of *FGFR2* gene amplification was higher in GC than in other types of cancer, consistent with previous studies [17, 22, 25] indicating an incidence of *FGFR2* amplification in GC of approximately 1.3%.

We also confirmed that the occurrence of *FGFR2* amplification was correlated with potential biomarkers, including MSI status and PD-L1 expression. FGFR2 has been reported to promote PD-L1 expression in a xenograft mouse model and induce PD-L1 expression via the JAK/STAT3 signaling pathway, causing apoptosis of T lymphocytes in CRC [37]. Consistent with a previous report [32], a strong correlation between *FGFR2* amplification and PD-L1 expression was observed in CBD cancer. However, we found that *FGFR2* amplification was unlikely to affect PD-L1 expression levels in gastric cancer, with a broad range of PD-L1 expression levels detected regardless of *FGFR2* copy number. Nevertheless, the results of correlation analysis for *FGFR2* gene amplification and PD-L1 should be interpreted with caution because samples comprising >57.1% of the total sample set were excluded (i.e., samples for which data were not available). Therefore, further studies are needed to elucidate the relationship between *FGFR2* amplification and PD-L1 expression.

Chromosomal rearrangement of the *FGFR2* gene is the most common type of *FGFR* gene fusion, with its frequency exceeding those of *FGFR1* and *FGFR3* [33]. It has been reported that *FGFR2* fusions occur with diverse fusion partners, especially in cholangiocarcinoma, and these genetic aberrations of *FGFR2* induce cancer cell proliferation and tumorigenesis [32–35]. Consistent with the literature [33, 35, 36], the results of the present study showed fusion of the *FGFR2* gene with nine different partners, namely, *TACC2*, *BICC1*, *BTBD16*, *WAC*, *HFM1*, *HOOK1*, *INPP5F*, *C10orf90*, and *WDR11*, in five different types of cancer. Among these fusions, *FGFR2*-*TACC2* and *FGFR2*-*BICC1* were observed in gastric and CBD cancers. Interestingly, *FGFR2* gene fusions are often correlated with high levels of PD-L1 expression in CBD cancer. In contrast, there was no correlation between *FGFR2* gene fusion and PD-L1 expression in gastric cancer. However, all cases of GC exhibited positive PD-L1 expression regardless of whether *FGFR2* genetic alterations were present (CPS, 1–10), suggesting that anti-PD-L1 therapy may be beneficial in the treatment of gastric cancer. Although there was a relatively weak correlation between *FGFR2* genetic alteration and the biomarker PD-L1, the blockade of *FGFR2* aberrations in human cancers is considered to be a promising approach for targeted therapy.

Based on our results and others [37, 38], FGFR2 IHC using FGFR2 antibody may not be a useful diagnostic utility to identify cancer patients with FGFR2 aberration in their tumor specimen. In the FIGHT trial, FGFR2b antibody was used as a screening method. However, FGFR2 antibody for the FIGHT trial was developed using their FGFR2b specific antibody [18] for patient screening.

It may be possible to treat advanced GC by regulating the FGFR signaling pathway, which may serve as a prognostic molecular target. Several reports have supported the efficacy of monoclonal antibodies targeting FGFR2, such as PRO-007 [11], AZD4547 [17, 22], futibatinib [39], and bemarituzumab [26], in reducing GC progression. In addition, AZD4547 was shown to exert antitumor activity in GC lines with *FGFR2* amplification [22].

Phase 1 and 2 clinical studies conducted in GC evaluating the efficacy of various inhibitors targeting FGFR have been reported [23–25]. In particular, pemigatinib, a small molecular inhibitor of FGFR, has shown efficacy and safety in patients with metastatic solid tumors with FGFR alteration in phase 2 and 3 clinical trials (FIGHT-201,202,302) [40–42] and has received accelerated approval in the USA for the treatment of metastatic CCA harboring FGFR2 gene rearrangements or fusions. This clinical evidence led to the design of another phase 2 trial in refractory, metastatic GC patients (the FiGhTeR trial) [43]. Bemarituzumab, a first-in-class humanized IgG2 monoclonal antibody plus first-line chemotherapy (oxaliplatin, 5-fluorouracil, leucovorin, and FOLFOX), demonstrated improved overall survival in patients with FGFR2^+^ GC [44].

## 4. Conclusion

In conclusion, we observed *FGFR2* aberrations, that is, gene amplification and fusion, within a cohort of Korean patients at our precision oncology clinic. NGS screening identified 21 patients as positive for *FGFR2* amplification (1.5%) and 15 patients as positive for *FGFR2* fusion (1.1%), with eight patients testing positive for both *FGFR2* amplification and fusion (0.6%). High levels of PD-L1 expression are more closely related to *FGFR2* fusion in CBD cancer. Besides, we found that patients with FGFR2 alteration had poorer OS and PFS than patients without FGFR2 alteration. Although further prospective studies in larger cohorts are required to determine the relationships between *FGFR2* aberrations and several potent biomarkers, including PD-L1 levels, there is accumulating evidence for the efficacy of therapeutic strategies involving the regulation of the FGFR signaling pathway, supporting the potential development of FGFR2-targeted therapy for precision medicine.

## 5. Materials and Methods

### 5.1. Patient Enrollment

The collection of specimens and associated clinical data used in this study was approved by the Institutional Review Board of Samsung Medical Center (IRB# 2021-09-052). All patients who participated in this study provided written informed consent prior to enrollment and specimen collection. This study was performed in accordance with the principles of the Helsinki Declaration and the Korean Good Clinical Practice guidelines.

### 5.2. Tumor DNA Extraction

Genomic DNA was acquired from formalin-fixed paraffin-embedded (FFPE) tissue sections (generally measuring 6–10 nm) and then purified using a commercial kit (Qiagen AllPrep DNA/RNA FFPE kit; Qiagen, Venlo, the Netherlands). The concentration of the purified DNA was determined using a Qubit DSDNA HS Assay kit (Thermo Fisher Scientific, Waltham, MA). Aliquots of 40 ng of DNA from each sample were used for DNA library preparation. The DNA integrity number (DIN) was obtained to determine the size of the DNA fragments, and the DNA quality was determined using the Genomic DNA ScreenTape assay (Agilent Technologies, Santa Clara, CA) on an Agilent 2200 TapeStation system (Agilent Technologies, Santa Clara, CA, USA).

### 5.3. Library Preparation, Sequencing, and Data Analysis

DNA libraries of all samples were prepared using a hybrid capture-based TruSight Oncology 500 (TSO 500) DNA/RNA Next Seq kit (Illumina, San Diego, CA, USA) in accordance with the manufacturer's protocol. Unique molecular identifiers (UMIs) to determine unique coverage at each position and increase accuracy during sequencing were used in the TSO 500, which allowed the detection of variants and variant allele frequency (VAF) while simultaneously suppressing errors, providing high specificity.

Sequence data were examined to identify clinically relevant classes of genomic alterations, including copy number variation (CNV) and rearrangements/fusions. An average CNV ≥ 4 was considered amplification and < 1 was considered a loss. Only gain was measured in the TSO 500 CNV files, and RNA translocation supporting reads > 4–12 is considered a translocation, subject to the quality of the sample. Filtered data exported from the TSO 500 pipeline [28] were annotated using the Ensembl Variant Effect Predictor (VEP) Annotation Engine [28] with information from databases, such as gnomAD genome and exome, 1000 genomes, dbSNP, COSMIC, RefSeq, ClinVar, and Ensembl. Genomic changes were categorized according to the 4-tier system proposed by the American Society of Clinical Oncology and the College of American Pathologists [29]. The TMB and MSI were recorded from the TSO 500 pipeline. In brief, the TMB detection is done by analyzing the following parameters: filtering any variant with an observed allele count ≥ 10 in any of the gnomAD exome, genome, and 1000 genomes databases, including variants in the coding region (RefSeq Cds) and variant frequency ≥ 5% of the total coding region with coverage > 50×. SNVs and indels: nonsynonymous and synonymous variant MSI status was determined using the calculated data from microsatellite sites relative to a set of normal baseline samples. The MSI score was determined by the percentage of unstable MSI sites to the total number of assessed MSI sites.

### 5.4. Immunohistochemistry (IHC)

GC FGFR2 IHC was performed using Benchmark XT (Ventana, Tucson, AZ, USA). Fixed tissues were embedded in paraffin blocks and sectioned at 3 *μ*m thickness. Each section was deparaffinized in xylene, and antigen retrieval was performed. Samples were incubated with anti-FGFR2 using a Dako Autostainer Link 48 (Agilent Technologies, Santa Clara, CA, USA). All IHC samples were scanned using a ScanScope Aperio AT Turbo slide scanner (Leica Microsystems, Melbourne, Australia).

### 5.5. Statistical Analysis

Overall survival (OS) period was defined as the time from initiation to chemotherapy until the date of death. Progression-free survival (PFS) was defined as the time from initiation to chemotherapy to the date of disease progression or all-cause mortality. Kaplan–Meier estimates were used in the analysis of all time to event variables, and the 95% confidence interval for the median time to event was computed. Survival analysis was performed using R for windows (version 4.1.2, https://cran.r-project.org/bin/windows/base/), and RStudio desktop 1.4 was used for drawing graphics (RStudio Team, 250 Northern Ave, Boston, MA 02210, USA; https://www.rstudio.com/products/rstudio/download/). Correlations between PD-L1 expression and the presence or absence of *FGFR2* genetic alterations were estimated using Pearson's correlation analysis using Prism 5 (GraphPad Software, USA).

## Data Availability

The data used to support the findings of this study are included within the article.

## References

[1] De Luca A., Esposito Abate R., Rachiglio A. M. (2020). FGFR fusions in cancer: from diagnostic approaches to therapeutic intervention. *International Journal of Molecular Sciences*.

[2] Hierro C., Alsina M., Sánchez M., Serra V., Rodon J., Tabernero J. (2018). Targeting the fibroblast growth factor receptor 2 in gastric cancer: promise or pitfall?. *Annals of Oncology*.

[3] Tiong K. H., Mah L. Y., Leong C. O. (2013). Functional roles of fibroblast growth factor receptors (FGFRs) signaling in human cancers. *Apoptosis*.

[4] Xie Y., Su N., Yang J. (2020). FGF/FGFR signaling in health and disease. *Signal Transduction and Targeted Therapy*.

[5] Fletcher M. N., Castro M. A., Wang X. (2013). Master regulators of FGFR2 signaling and breast cancer risk. *Nature Communications*.

[6] Giacomini A., Grillo E., Rezzola S. (2021). The FGF/FGFR system in the physiopathology of the prostate gland. *Physiological Reviews*.

[7] Krook M. A., Reeser J. W., Ernst G. (2021). Fibroblast growth factor receptors in cancer: genetic alterations, diagnostics, therapeutic targets and mechanisms of resistance. *British Journal of Cancer*.

[8] Deng N., Goh L. K., Wang H. (2012). A comprehensive survey of genomic alterations in gastric cancer reveals systematic patterns of molecular exclusivity and co-occurrence among distinct therapeutic targets. *Gut*.

[9] Dienstmann R., Rodon J., Prat A. (2014). Genomic aberrations in the FGFR pathway: opportunities for targeted therapies in solid tumors. *Annals of Oncology*.

[10] Gill C. M., Orfanelli T., Yoxtheimer L. (2020). Histology-specific FGFR2 alterations and FGFR2-TACC2 fusion in mixed adenoid cystic and neuroendocrine small cell carcinoma of the uterine cervix. *Gynecologic Oncology Reports*.

[11] Kim S., Dubrovska A., Salamone R. J. (2013). FGFR2 promotes breast tumorigenicity through maintenance of breast tumor-initiating cells. *PLoS One*.

[12] Matsumoto K., Arao T., Hamaguchi T. (2012). *FGFR2* gene amplification and clinicopathological features in gastric cancer. *British Journal Of Cancer*.

[13] Turner N., Lambros M. B., Horlings H. M. (2010). Integrative molecular profiling of triple negative breast cancers identifies amplicon drivers and potential therapeutic targets. *Oncogene*.

[14] Cha Y., Kim H. P., Lim Y., Han S. W., Song S. H., Kim T. Y. (2018). FGFR2 amplification is predictive of sensitivity to regorafenib in gastric and colorectal cancers in vitro. *Molecular Oncology*.

[15] Hur J. Y., Chao J., Kim K. (2020). High-level FGFR2 amplification is associated with poor prognosis and lower response to chemotherapy in gastric cancers. *Pathology-Research and Practice*.

[16] Kim S. T., Lee I. K., Rom E. (2019). Neutralizing antibody to FGFR2 can act as a selective biomarker and potential therapeutic agent for gastric cancer with FGFR2 amplification. *American Journal of Translational Research*.

[17] Xie L., Su X., Zhang L. (2013). FGFR2 gene amplification in gastric cancer predicts sensitivity to the selective FGFR inhibitor AZD4547. *Clinical Cancer Research*.

[18] Ahn S., Lee J., Hong M. (2016). FGFR2 in gastric cancer: protein overexpression predicts gene amplification and high H-index predicts poor survival. *Modern Pathology*.

[19] Katoh Y., Katoh M. (2009). FGFR2-related pathogenesis and FGFR2-targeted therapeutics (review). *International Journal of Molecular Medicine*.

[20] Peters K. G., Werner S., Chen G., Williams L. T. (1992). Two FGF receptor genes are differentially expressed in epithelial and mesenchymal tissues during limb formation and organogenesis in the mouse. *Development*.

[21] Zhang J., Tang P. M. K., Zhou Y. (2019). Targeting the oncogenic FGF-FGFR axis in gastric carcinogenesis. *Cell*.

[22] Jang J., Kim H. K., Bang H. (2017). Antitumor effect of AZD4547 in a fibroblast growth factor receptor 2-amplified gastric cancer patient-derived cell model. *Translational Oncology*.

[23] Hollebecque A., Doi T., Saavedra O. (2020). A phase II study of futibatinib (TAS-120) in patients (pts) with advanced (adv) solid tumors harboring fibroblast growth factor receptor (FGFR) genomic aberrations. *Journal of Clinical Oncology*.

[24] Michael M., Bang Y. J., Park Y. S. (2017). A phase 1 study of LY2874455, an oral selective pan-FGFR inhibitor, in patients with advanced cancer. *Targeted Oncology*.

[25] Van Cutsem E., Bang Y. J., Mansoor W. (2017). A randomized, open-label study of the efficacy and safety of AZD4547 monotherapy versus paclitaxel for the treatment of advanced gastric adenocarcinoma with *FGFR2* polysomy or gene amplification. *Annals of Oncology*.

[26] Catenacci D. V. T., Rasco D., Lee J. (2020). Phase I escalation and expansion study of bemarituzumab (FPA144) in patients with advanced solid tumors and FGFR2b-selected gastroesophageal adenocarcinoma. *Journal of Clinical Oncology*.

[27] Kim H., Hong J. Y., Lee J. (2021). Clinical sequencing to assess tumor mutational burden as a useful biomarker to immunotherapy in various solid tumors. *Therapeutic Advances in Medical Oncology*.

[28] Pestinger V., Smith M., Sillo T. (2020). Use of an integrated pan-cancer oncology enrichment next-generation sequencing assay to measure tumour mutational burden and detect clinically actionable variants. *Molecular Diagnosis & Therapy*.

[29] Karasaki T., Nakajima J., Kakimi K. (2016). Neoantigens and whole-exome sequencing. *Gan to Kagaku Ryoho*.

[30] Qin A., Johnson A., Ross J. S. (2019). Detection of known and novel FGFR fusions in non-small cell lung cancer by comprehensive genomic profiling. *Journal of Thoracic Oncology*.

[31] Zhang J., Wong C. C., Leung K. T. (2020). FGF18-FGFR2 signaling triggers the activation of c-Jun-YAP1 axis to promote carcinogenesis in a subgroup of gastric cancer patients and indicates translational potential. *Oncogene*.

[32] Arai Y., Totoki Y., Hosoda F. (2014). Fibroblast growth factor receptor 2 tyrosine kinase fusions define a unique molecular subtype of cholangiocarcinoma. *Hepatology*.

[33] Helsten T., Elkin S., Arthur E., Tomson B. N., Carter J., Kurzrock R. (2016). The FGFR landscape in cancer: analysis of 4,853 tumors by next-generation sequencing. *Clinical Cancer Research*.

[34] Borad M. J., Champion M. D., Egan J. B. (2014). Integrated genomic characterization reveals novel, therapeutically relevant drug targets in FGFR and EGFR pathways in sporadic intrahepatic cholangiocarcinoma. *PLoS Genetics*.

[35] Lowery M. A., Ptashkin R., Jordan E. (2018). Comprehensive molecular profiling of intrahepatic and extrahepatic cholangiocarcinomas: potential targets for intervention. *Clinical Cancer Research*.

[36] Wu Y. M., Su F., Kalyana-Sundaram S. (2013). Identification of targetable FGFR gene fusions in diverse cancers. *Cancer Discovery*.

[37] Li P., Huang T., Zou Q. (2019). FGFR2 promotes expression of PD-L1 in colorectal cancer via the JAK/STAT3 signaling pathway. *Journal of Immunology*.

[38] Hosoda K., Yamashita K., Ushiku H. (2018). Prognostic relevance of FGFR2 expression in stage II/III gastric cancer with curative resection and S-1 chemotherapy. *Oncology Letters*.

[39] Sootome H., Fujita H., Ito K. (2020). Futibatinib is a novel irreversible FGFR 1-4 Inhibitor that shows selective antitumor activity against FGFR-deregulated tumors. *Cancer Research*.

[40] Necchi A., Pouessel D., Leibowitz-Amit R. (2018). Interim results of fight-201, a phase II, open-label, multicenter study of INCB054828 in patients (pts) with metastatic or surgically unresectable urothelial carcinoma (UC) harboring fibroblast growth factor (FGF)/FGF receptor (FGFR) genetic alterations (GA). *Annals of Oncology*.

[41] Bekaii-Saab T. S., Valle J. W., Van Cutsem E. (2020). FIGHT-302: first-line pemigatinib vs gemcitabine plus cisplatin for advanced cholangiocarcinoma withFGFR2rearrangements. *Future Oncology*.

[42] Abou-Alfa G. K., Sahai V., Hollebecque A. (2021). Pemigatinib for previously treated locally advanced/metastatic cholangiocarcinoma (CCA): update of FIGHT-202. *Journal of Clinical Oncology*.

[43] Merz V., Zecchetto C., Simionato F. (2020). A phase II trial of the FGFR inhibitor pemigatinib in patients with metastatic esophageal-gastric junction/gastric cancer trastuzumab resistant: the FiGhTeR trial. *Therapeutic Advances in Medical Oncology*.

[44] Catenacci D. V. T., Kang Y.-K., Saeed A. (2021). FIGHT: a randomized, double-blind, placebo-controlled, phase II study of bemarituzumab (bema) combined with modified FOLFOX6 in 1L FGFR2b+ advanced gastric/gastroesophageal junction adenocarcinoma (GC). *Journal of Clinical Oncology*.

